# Trauma and Growth: Impact of AIDS Activism

**DOI:** 10.1155/2018/9696725

**Published:** 2018-05-24

**Authors:** Judith G. Rabkin, Martin C. McElhiney, Mark Harrington, Tim Horn

**Affiliations:** ^1^New York State Psychiatric Institute, New York, NY, USA; ^2^Department of Psychiatry, Columbia University, New York, NY, USA; ^3^Treatment Action Group, New York, NY, USA

## Abstract

**Introduction:**

Our goal was to assess the long-term impact of AIDS activism of ACT UP/New York on the current adjustment of those who were members during its peak years (1987–1992), including assessment of trauma sequelae as well as posttraumatic growth.

**Methods:**

A 90-minute semistructured interview and 6 validated self-report scales were administered. We relied on purposive and snowball sampling to recruit potential participants. Areas covered include demographics, ACT UP participation, and psychiatric problems. Self-report scales provided approximate diagnoses of PTSD and depression, as well as coping, optimism, and related concepts.

**Results:**

Participants included 102 men (40% HIV-positive) and 23 women. Seventeen percent reported current symptoms suggesting PTSD, slightly above the range in general population studies. Symptoms consistent with depression were reported by 8% overall, with higher rates for HIV+ men. Enhanced sense of self, belief in change, and empowerment were reported by 93% of respondents, independent of concurrent PTSD or depression.

**Conclusions:**

Twenty-eight years later, ACT UP study participants recall their activist days during the AIDS epidemic as the peak experience of their lives. While some continue to have symptoms of stress and depression, most found that their activism has enriched their subsequent lives.

## 1. Introduction

In the early years of the HIV/AIDS epidemic in the United States, activists played a unique and unprecedented role. Confronting a new, lethal, and highly stigmatized disease, they became caregivers, advocates, “citizen scientists,” and the voice of an outraged community [1]. While the history of organizations such as ACT UP (the AIDS Coalition to Unleash Power) continues to be documented [2], the long-term psychosocial effects of AIDS activism on activists themselves have yet to be well studied. What happens when members of a marginalized community, threatened both by a rapidly fatal disease and by an unresponsive government, organize to fight for survival and survive? We examined the prevalence of posttraumatic stress disorder (PTSD), depression, and posttraumatic growth among men and women who participated in ACT UP between 1987 and 1992.

ACT UP was founded in 1987 in New York City as a radical protest organization; peak membership and activity occurred between 1987 and 1992. Its members were exposed to extensive loss and trauma because of the intensity of their work, the high mortality within their extended networks of HIV-exposed fellow activists and friends, their own risk of illness and death, and their confrontational demonstrations. At the same time, they had the opportunity to connect with a network, find emotional support, and act on behalf of their own and their community's lives. ACT UP survivors thus constitute a distinctive sample with which to address key questions of long-term trauma and growth.

ACT UP members were predominantly young, gay, and often but not always HIV-infected, although women (often but not always lesbian) also played leading roles. ACT UP members conducted major, very visible demonstrations, starting within a month of its formation by Larry Kramer in March 1987. In September of that year, a demonstration took place inside the New York Stock Exchange, protesting the high cost of AZT, the only medication at that time approved to treat AIDS. Few people with AIDS had health insurance or access to medical care and were sometimes evicted, fired, and rejected by their families. ACT UP had committees whose members pioneered innovative housing for people with AIDS, piloted syringe exchanges and other harm reduction projects for drug users, and worked with the FDA and NIH-funded AIDS trial networks, to plan drug trials and facilitate medication access at a time when no effective treatments were available. ACT UP was loud; it was confrontational; it was effective.

The long-term effects of ACT UP participation are unclear. Those who were engaged in the years 1987–1992 today are all “long-term” survivors of the epidemic years and, for some, survivors with HIV/AIDS. Some have proposed that community activism has a protective effect, often leading to posttraumatic growth [3], while others such as Spencer Cox (“Living on the Edge: Gay men in mid-life, and the impact of HIV/AIDS,” unpublished) emphasized negative outcomes including posttraumatic stress disorder, clinical depression, and substance use.

Following the Cox initiative, we included measures of posttraumatic stress disorder, major depression, and substance use to assess long-term negative sequelae of ACT UP participation, acknowledging that neither we nor respondents would be able to rule out the concurrent effects of living in the midst of the AIDS epidemic itself. Major depressive disorder has been recognized as a significant psychiatric problem since the development of formal diagnostic criteria. PTSD has been an official psychiatric disorder since 1980 [4], although the concept of shell shock in combat veterans has a much longer history. The formal definition of PTSD requires exposure to “actual or threatened death, serious injury, or sexual violence, either by direct experience, witnessing, learning that such an event occurred to someone close, or experiencing repeated exposure to aversive details of the traumatic event” [5], along with associated symptoms such as numbness, intrusive recollections, and hyperarousal. Symptom duration varies. Among American soldiers deployed in Iraq assessed 7 years later and Romanian political prisoners 40 years later, 25–30% still had PTSD symptoms [6, 7]. For both PTSD and depression, diagnosis requires clinically significant distress or impairment in major areas of functioning.

In addition to formal psychiatric diagnoses such as depressive disorder and PTSD, the lay term “AIDS Survivor Syndrome” (ASS) is defined by its proponents as a “spectrum of sustained trauma of survivorship, the psychological state resulting from living through the HIV/AIDS pandemic” [8]. It is intended to represent a different phenomenon than PTSD and is conceptualized as a normal response to an exceptional experience [9].

Some survivors of the earliest days of the AIDS epidemic continue to report ongoing distress and “long-term survivor” support groups continue to exist. Overall, however, the limited evidence, largely published in the early 1990s, reflects the prominence of resiliency over pathology [10–13]. In the general population, an estimated 60% to 80% of those who endure traumatic or highly stressful events do not develop psychiatric disorders [14, 15].

Protective conditions, relations, or circumstances have been examined in various contexts. A major factor is “unit cohesion.” Junger [16] observed that membership in tribes and communities provides a powerful protective effect during wartime, noting that admissions to psychiatric hospitals actually declined during the London Blitz of 1940-41 and the Dutch famine of 1944-45. The same has been observed in wartime among elite frontline troops most directly exposed to danger, who have lower rates of psychiatric breakdown compared to rear-base troops. “Unit cohesion,” strong emotional bonds of friendship, and mutual trust within a community, company, or platoon are considered to protect against otherwise virtually intolerable experiences [17].

Although the negative effects of trauma have been studied for years, the concept of “posttraumatic growth” [18, 19] and the study of “positive psychology” have evolved more recently [20]. Folkman [21] added the concept of positive reappraisal to her model of stress and coping, in which the significance of a stressful event is reinterpreted in a positive way. Reappraisal is similar to cognitive reframing, a standard component of cognitive behavioral therapy [22]. PTSD and posttraumatic growth can be conceptualized, not as poles of one continuum, but as two independent constructs that can coexist.

Building on these observations, we assessed the current rates of PTSD, depression, and substance abuse, as well as posttraumatic growth, among ACT UP survivors. We explored their association with exposure to losses and other experiences associated with AIDS activism. We attempted to determine the extent to which the alternative model of AIDS Survivor Syndrome overlaps with measures of depression and PTSD. We also evaluated the protective effects of “unit cohesion” and posttraumatic growth with respect to sense of self, relationships, and life outlook.

## 2. Methods

### 2.1. Sampling and Recruitment

ACT UP New York drew several hundred members to its weekly meetings in its peak years [31], but there was no official membership list and no way to obtain a random sample of survivors. Instead, we used purposive sampling followed by snowball sampling to identify study participants. We relied on Community Advisory Board members to contact their social networks and to post notices about the study on the ACT UP Alumni Facebook page to recruit volunteers. Controls (defined as those present in NYC between 1987 and 1992 who identified with the lesbian/gay community but who chose not to join ACT UP) were identified by ACT UP study participants.

### 2.2. Measures

Study tools were selected and a semistructured interview was developed following preliminary discussions with seven key informants (six were prominent in HIV research or treatment including two psychiatrists, and one was a former HIV+ activist). We asked about experiences during ACT UP participation as well as past and current medical and psychological problems. Participants completed six validated self-report scales [23–30] assessing current coping, optimism, loneliness, depression and PTSD symptoms and DSM-IV diagnoses, and alcohol use (Table 1). Algorithms for the measures of PTSD (PCL using Ruggiero's [26, p. 500] preferred method of deriving DSM-IV diagnosis) and depression (PHQ) were used to determine approximate DSM-IV diagnoses; formal psychiatric interviews were not conducted. Continuous scores on the PCL and PHQ also were used.

### 2.3. Data Collection

Interviews lasted about 90 minutes and took place between June 2013 and March 2015. Local participants were interviewed in person, while those outside New York City were interviewed by telephone. Interviews were audio-taped. Structured questions were coded, and open-ended queries were transcribed.

### 2.4. Data Analysis

Three open-ended queries were coded by question and theme. They inquired about “best things” about ACT UP, “worst things,” and “sense of self.” Three team members rated each and met to reach consensus on classification. We analyzed quantitative data using* t*-tests, chi-square for categorical variables, and Pearson's correlations. Fisher's Exact Test (FET) was used for analysis of categorical variables when cell size < 6. Three separate multiple regression analyses were performed, using total scores of each as the dependent variable to identify factors associated with depression, PTSD, and coping effectiveness. All predictors were included in the model simultaneously. Skewed data were log-transformed. Since this is an exploratory study, we did not correct for multiple comparisons but did not report statistical trends (*p* > .05 − < .10). We defined statistical significance as *p* ≤ .05, 2-tailed, in all analyses.

### 2.5. Ethical Approvals

The study was approved by the Institutional Review Board of New York State Psychiatric Institute-Columbia University. Participants signed consent forms after the consent process was conducted and all questions were addressed.

## 3. Results

### 3.1. Sample Characteristics

ACT UP members between 1987 and 1992 (102 men and 23 women) and 50 controls (non-ACT UP members) were study participants (Table 2). Seven continued to be ACT UP/New York members. Among members, 41 men and no women were HIV+. Nineteen of 50 controls were HIV+; all were male. Two-thirds of members and all controls were interviewed in New York City; the remaining 33 participants lived in 14 states and three foreign countries. Among members at the time of the interview, the mean age was 54 years (range: 37–74); 85% had completed college including 47% who completed graduate school. Thirty-six percent completed their education after leaving ACT UP. Three-quarters (76%) reported annual income of over $30,000 and 40% earned over $75,000 per year. Ninety-eight percent of participants were identified as gay/lesbian. Controls did not differ on these demographic measures.

### 3.2. Rates and Correlates of PTSD

Twenty-one respondents (17%) reported current symptoms consistent with an approximate DSM-IV diagnosis of PTSD, including 20 men, of whom half were HIV-positive, and one woman. Comparing participants with and without PTSD (Table 3(a)), we found no differences in current age, income, education, or HIV serostatus. In addition, no differences were found in duration of ACT UP participation, number of demonstrations, arrests, frequency of attending, or other measures of activism. However, those with PTSD reported more deaths of friends (*p* = .049) and scored significantly worse on all self-reported measures of current adjustment and functioning (all *p* values < 0.002) (Table 3(b)). In a regression analysis (Table 4(a)) using total PCL score as outcome, older age (*p* = .02) and committee membership during ACT UP (*p* = .04) were associated with lower scores, while those having intrusive thoughts and lacking future orientation were more likely to have higher scores (more PTSD symptoms, *p* < .001 and *p* = .03, respectively).

### 3.3. Prevalence of Depression

The depression measure, PHQ, provides both an* approximate* DSM-IV diagnosis of depressive disorders and a numerical indicator of symptom severity. HIV status is associated with current major depressive disorder (MDD), with nine HIV+ men and one HIV− man having a major depressive disorder. In a regression analysis using variables listed in Table 4(b), those with close friends (when interviewed) were 51% less likely to be depressed than others (*t* = −3.92, *p* < .001), adjusting for all covariates.

Of note, there was substantial overlap among participants with approximate diagnoses of depression (PHQ) and PTSD (PCL): for the entire sample, the correlation between scores on the PHQ and PCL was 0.74 (*p* < .001). We reanalyzed PCL scores after deleting three items (difficulty sleeping, problems with concentration, and loss of interest in formerly pleasurable events) that correspond to criteria for depressive disorders and prorated scores for the missing items. The correlation between the PHQ and adjusted PCL was 0.70 (*p* < .001), remaining essentially the same.

A small number of respondents (5 on the PCL and 1 on the PHQ) had high scores on these measures (over the cut-off on the PCL of 50 and on the PHQ of 14, used to signify a probable diagnosis). However, the distribution of their responses did not conform to the diagnostic algorithms of the respective measures, reflecting substantial distress without a diagnosis.

### 3.4. The Role of Substance Use

We asked respondents about current (past year) and historical use of recreational drugs and alcohol with open-ended questions. When reported, was there interference with social or occupational functioning? If yes, we defined this use as a “problem.” Within the past year, 11 respondents (9%) reported problems with alcohol, methamphetamine, marijuana, and/or cocaine, in descending order of frequency. Of these, three had concurrent PTSD and/or depression.

Fifty-five respondents (44%) reported past substance use problems. The most common was alcohol, reported by 36 respondents (29%), including 11 HIV-positive men (27%), 20 HIV-negative men (33%), and 5 women (22%). Two-thirds had started drinking heavily before they joined ACT UP. Of the 15 men (no women) who reported past methamphetamine problems, 11 were HIV-positive. Prior marijuana problems, reported by 14 respondents, and cocaine problems, reported by 12, did not differ by gender or HIV serostatus.

### 3.5. Impact of HIV Serostatus

When interviewed, 40% (41/102) of the men were HIV-positive. Ten (25%) had been diagnosed with HIV prior to ACT UP, and a total of 32 (78%) by 1995, when effective combination antiretroviral treatment became available. HIV-positive men did not differ from HIV seronegative men on demographic measures, scales assessing coping, loneliness, alcohol use, or optimism, nor did they differ in terms of ACT UP experiences, with one exception: more reported loss of a partner (Table 5). In terms of current psychological adjustment, they were more likely to have a current major depressive disorder (9/41 = 22% versus 1/61 = 2%, FET ≤ 0.003) and reports of past methamphetamine substance use disorder (11/15, FET ≤ .001).

### 3.6. AIDS Survivor Syndrome

Manifestations include depression, anger, anxiety, emotional numbness, behavior (e.g., sexual risk-taking, isolation), hopelessness, lack of future orientation, and social isolation. We identified 16 measurable symptoms for which we had data, excluding concepts such as “panic from unexpected older age” and “personality changes,” and examined their distributions. The mean number of items affirmed by the entire sample was 2.4 (range: 0–13). For the 21 respondents with PTSD (PCL Scale), the mean number of ASS items endorsed was 6.8 (SD = 2.98), significantly greater than for the rest of the sample (*t* = 7.77, *p* < .001). For the 10 respondents classified as depressed (PHQ Scale), mean number was 8.7 (SD = 2.90) items endorsed, again greater than the rest of the sample (*t* = −7.163, *p* < .001). Looking at the symptom distribution for the sample (*N* = 125), the lowest quartile expressed no symptoms, while the mean for the top quartile was 6.4 (SD = 2.5).

### 3.7. “Unit Cohesion”

When asked “In what ways was ACT UP a positive experience?,” 99 respondents (79%) cited “social ties,” the most commonly endorsed choice among 10 options provided (respondents could and usually did endorse more than one; see the Appendix). Other frequently endorsed (and overlapping) response options were “collective identity” (*N* = 85, 68%) and “emotional support” (*N* = 54, 43%).

When asked whether they missed anything after leaving ACT UP, with seven response options (see the Appendix), the most commonly endorsed was “loss of network.” Finally, when asked to list the two “best things” about ACT UP in an open-ended format, the most commonly mentioned “best thing” was “sense of community, being part of something” (*N* = 45, 37%). For example, one man said, “It was an all-encompassing sense of family and support. It was my family, my church, my sex life, where I got my optimism.” Another said, “an incredible sense of camaraderie, being part of an army trying to change the world.” When asked about the two “worst things,” the most common responses were losses, “all the deaths that kept accelerating, without time for the grief process,” and the infighting and arguments at meetings, “the acrimony, internal discord.” Both weakened unit cohesion.

### 3.8. Posttraumatic Growth: Change in Sense of Self

In response to the open-ended query, “in what ways was ACT UP a positive experience?,” 65% of the respondents described the theme of “empowerment.” One respondent noted, “I was identified as an AIDS activist, a different identity that was strong and tough. I felt at the point of a spear.” Another reflected, “I had agency. I could do something” and “the life and death context brought out the best in me. I could do something that was bigger than me and changed the world.”

Respondents were asked, “Do you think your experience in ACT UP changed your sense of self, who you are as a person?” The overwhelming response was affirmative (93%), regardless of whether or not PTSD symptoms were present 25 years later.  Table 6 presents a sample of responses for the three most common categories: agency (*N* = 32), empowerment (*N* = 28), and personal growth (*N* = 29). Other themes, endorsed by less than 5% of respondents, were future life choices, a new identity, and finding meaning. One respondent illustrated parallel feelings of benefit and then loss: “I believe people in ACT UP experienced something really extraordinary and that people who have fought the good fight have the problem that when the fighting stops…there's a problem of finding meaning in ordinary life and rejoining the real world which really isn't compelling.”

In a regression analysis (Table 4(b)), using the Coping Self-Efficacy Scale as the dependent variable, better coping was associated with minority status, having close friends, and those with partners, while holding all other covariates constant.

## 4. Discussion

Our findings support the notion of posttraumatic stress responses and posttraumatic growth as independent, parallel dimensions rather than endpoints in a single continuum. Taylor's [32] cognitive adaptation theory proposes that the adjustment to traumatic events includes three components: search for meaning (also cited in Folkman's expansion of her theory of stress and coping [21]), an attempt to regain control, and an effort to improve self-esteem. All three of these themes are expressed in responses (Table 6) to our query about the impact of ACT UP on sense of self.

We found somewhat elevated prevalence of both PTSD and depression in this sample of AIDS activists 25 years later, compared to rates found in general population-based studies, which range from 1% to 14% ([5], p. 426). Of note, the relationship of traumatic symptoms with ACT UP experiences is at best an estimate. While respondents were instructed to complete the PCL Checklist referring* only* to AIDS or ACT UP-related trauma, two-thirds reported traumatic events that were* not* specific to either.

The overall rate of major depressive disorder in our sample was 8%, with higher rates for HIV+ men. The prevalence of depression is known to be elevated among HIV+ men and women [33]. In national studies of HIV-positive adults, the observed rates of major depression range from 5.9% [34] to 22% [35], compared to 7% in the general population [36]. Although our finding for HIV+ men is at the high end of this range, it may be a measurement artifact: we relied only on self-report, while the cited studies used formal psychiatric evaluations which usually generate lower rates than self-report scales.

The overlap between depression and PTSD is substantial in our study, as is widely recognized in the literature. As Shalev et al. noted [37], the concurrence of depression and PTSD is up to 56%. The causal relationship between the two is unclear since each can be a risk factor for the other [38]. Neumeister [39] reported that PTSD appears to be “an umbrella diagnosis for a broad spectrum of symptoms ranging from chronic anxiety and panic disorder to emotional numbing and depression.”

These diagnostic classifications did not include a subset of people who expressed stress-related or mood-related distress at a level below the diagnostic threshold. Since such findings are not formally reported, it is difficult to know whether the observed rates are common in other populations. Perhaps this is what is picked up by the ASS concept and may motivate some people to join support groups for long-term survivors.

Turning now to positive effects, participants found that ACT UP membership in 1987–1992 dramatically contributed to positive growth, which they still experience 25 years later. The vast majority reported an enhanced sense of self, confidence, belief in change, and their ability to influence events (agency). For some, there is also a sense of lingering, diffuse distress, not fully captured either by diagnoses of depression or PTSD: a sense of loss and echoes of distressing experiences. Loss refers not only to the deaths of friends and fellow ACT UP members, but also to loss of the spirit, the focus, and the support of ACT UP membership in its peak years. Respondents also spoke of loss of a sense of purpose, of relevance, of friendships and connection, the excitement and stimulation, the sense of community after the ACT UP years.

The concept of unit cohesion contributes to our understanding of the ability of ACT UP members to persevere in the face of unremitting AIDS losses, public and government indifference or outright hostility, and the intense confrontations including arrests that demonstrations entailed. In part, this cohesion derived from the organization within ACT UP, including committees and affinity groups that worked long hours together to plan and prepare for demonstrations and protests. In addition, standing Monday evening meetings, attended by all members, fostered a special identity and sense of belonging. Despite the grief, fear, anxiety and anger of the ACT UP years, the general sense of the 125 respondents is that these years were the highlight of their lives, the most important and influential experience they have had. Our findings suggest that group support is effective in achieving and maintaining well-being, with current implications for service interventions for long-term survivors of the epidemic generally, not only former ACT UP members.

More generally, activism in many contexts has been found to promote positive, increased self-esteem and well-being, as discussed by Klar and Kasser [3]. They suggest potential mediators such as the utilization of problem-focused coping [12] or heightened consistency between values and actions.

## 5. Study Strengths and Limitations

To our knowledge, this is the only study exploring the long-term impact of AIDS activism; its sample size and robust data collection make it a valuable contribution to the literature. Limitations include nonrandom sampling, although the demographics of our sample are roughly equivalent to those noted in a 1989 survey of 413 ACT UP/NY members conducted by Elbaz [40] in which 92% were white and 20% were women. Study participants may not represent the full spectrum of distress and disability since those who are doing better may be more likely to volunteer.

In conclusion, AIDS activists recalled their ACT UP years as “the best of times, the worst of times.” Dramatic personal growth was often accompanied by lingering sadness, unresolved grief, and, for some, loss of perceived life purpose. However, the community bonds within ACT UP played a central and protective role, enabling most members to move forward and lead productive lives. As one man remembered, “ACT UP was exciting, joyous, a strange intersection of elation at the time, providing a scaffolding over this chasm of grief and sorrow.”

## Figures and Tables

**Table 1:** Study measures: items, content, score range, and cut-offs (if applicable)^*∗*^.

Scale	Number of items	Content	Score range^*∗*^	Cut-offs
Patient Health Questionnaire (PHQ-9) (depression) [23]	9	Consists of the 9 criteria for diagnosis of major depression and minor depression (DSM-IV and 5); provides a provisional diagnosis and also a symptom severity score. Diagnosis of major depression requires ratings of “most days” (3) or “almost every day” (4) on 5+ items including either depressed mood or loss of interest. Minor depression requires only 3 items. Time frame: past 2 weeks.	0–27	<10 = absent/mild 10–14 = moderate 15–19 = moderately severe ≥20 = severe Diagnosis: algorithm

PTSD Checklist (PCL) [24–26]	17	Consists of DSM-IV symptoms of PTSD. It generates an approximate diagnosis, using an algorithm that requires 1+ symptoms scored 3+ on individual items from each of 3 symptom clusters. Time frame: past month.	17–85	>50 = “case” Diagnosis: algorithm

Alcohol Use Disorders Identification Test (AUDIT) [27]	10	Questions about frequency and amount plus possible negative consequences and regrets. Time frame: past year.	0–40	16–19 = mild problem ≥20 = suggests need for treatment

^*∗∗*^Coping Self-Efficacy Scale [28]	13	Measures perceived ability to cope effectively “when you're having problems.” We used 13 of the original 26 items. Example: “Sort out what can be changed and what cannot be changed.” No time frame.	0–130	None

Life Orientation Test (LOT) [29]	12	Designed to measure optimism versus pessimism. Example: “In uncertain times, I usually expect the best.” No time frame.	0–48	≥17 = optimism <17 = pessimism

^*∗∗*^UCLA Loneliness Scale [30]	10	Ten of the original 20 items, including both negative (“I feel isolated from others”) and positive (“There are people I can talk to”) items, the latter scored in reverse. Time frame: past 2 weeks.	10–40	None

^*∗*^ *Note*. Higher scores represent a greater endorsement of the construct being measured. ^*∗∗*^*Note*. Score doubled to enable comparison with norms.

**Table 2:** Demographic characteristics of ACT UP group and control group.

	ACT UP group				Control group *N* = 50
	Male HIV+ *N* = 41	Male HIV− *N* = 61	Female^*∗*^ *N* = 23	Total *N* = 125	
Age, M (range)	55 (45–73)	54 (44–74)	52 (37–68)	54 (37–74)	56 (44–68)
Ethnicity, *N* (%)					
White	35 (85)	51 (84)	20 (87)	106 (85)	44 (88)
Black	0	2 (3)	3 (13)	5 (4)	3 (6)
Hispanic	6 (15)	7 (11)	0	13 (10)	1 (2)
Other	0	1 (2)	0	1 (1)	2 (4)
Education (years), M (SD)	17.2 (2.3)	17.1 (2.3)	17.7 (2.5)	17.2 (2.3)	16.9 (2.2)
Education, *N* (%)					
Less than college	7 (17)	9 (15)	3 (13)	19 (15)	6 (12)
College	15 (37)	25 (38)	7 (30)	47 (38)	24 (48)
Graduate training	19 (46)	27 (44)	13 (57)	59 (47)	20 (40)
Income, *N* (%)					
Under $30,000	11 (28)	13 (22)	5 (22)	29 (24)	7 (14) 4 (8) 9 (18) 1 (2) 29 (58)
30,000–75,000	14 (35)	22 (37)	9 (39)	45 (36)	
76,000–99,999	4 (10)	11 (18)	3 (13)	18 (15)	
$100,000+	11 (27)	14 (23)	6 (26)	31 (25)	
Major depression diagnosis, *N* (%)	9 (22)	1 (2)	0	10 (8)	1 (2)

^*∗*^All HIV−.

**(a):** 

Demographic variables and ACT UP experiences	PTSD diagnosis *N* = 21	No diagnosis *N* = 104	*t* or *x*^2^	*p*
Current age, *M (SD)*	51.5 (4.9)	54.1 (6.6)	−1.730	.086
Current annual income, *M (SD)*	$76,176 (67,780)	$81,400 (106,326)	−.216	.829
Education (years), *M (SD)*	16.7 (2.5)	17.3 (2.3)	−.982	.328
HIV status, *N (%)*			2.326	.127
HIV+	10 (48)	31 (30)		
HIV−	11 (52)	73 (70)		
Lost friends, *N (%)*			6.051	.049
None	1 (5)	8 (8)		
Some	1 (5)	29 (28)		
Many	19 (90)	65 (63)		
Lost partner, *N (%)*	7 (33)	19 (18)	2.673	.102
Primary caregiver for sick friends, *N (%)*	8 (38)	29 (28)	.773	.379
Age when joined ACT UP, *M (SD)*	27.1 (4.5)	28.7 (6.1)	−1.155	.250
Number of years in ACT UP, *M (SD)*	7.1 (7.9)	5.0 (4.3)	1.670	.097
Demonstrations, *N (%)*				
None	-	2 (2)		
A few	1 (5)	1 (1)	2.626	.453
Some	1 (5)	11 (11)		
Many	19 (90)	88 (85)		
Times arrested, *N (%)*			.201	.904
None	7 (33)	30 (29)		
Once	2 (10)	12 (12)		
More than once	11 (52)	57 (55)		
Attended Monday meetings, *N (%)*				
Rarely	-	1 (1)		
Occasionally	1 (5)	5 (5)	.385	.943
Often	1 (5)	3 (3)		
Regularly	19 (90)	93 (89)		
In a committee, *N (%)*	14 (67)	85 (82)	3.080	.079

**(b):** 

Self-reported measures	PTSD diagnosis *N* = 21	No diagnosis *N* = 104	*t* or *x*^2^	*p*
PHQ-9 [depression], *M (SD)*	13.0 (5.7)	3.8 (3.3)	10.101	<.001
PHQ-9: DSM IV meets algorithm for major depression, *N (%)*	7 (33)	3 (3)	21.536	<.001
PCL Checklist (PTSD), *M (SD)*	54.7 (7.2)	29.1 (9.5)	11.633	<.001
PCL Checklist (PTSD): prorated total minus three depression items, *M (SD)*	53.8 (7.8)	28.4 (9.6)	11.330	<.001
AUDIT: problematic alcohol use (scores ≥ 16), *N (%)*	6 (29)	14 (13)	2.819	.093
Coping Self-Efficacy Scale, *M (SD)*	57.9 (16.9)	92.6 (18.4)	−7.980	<.001
LOT [optimism versus pessimism]			13.134	<.001
Optimistic (scores ≥ 17), *N (%)*	7 (33)	77 (74)		
Pessimistic (scores < 17), *N (%)*	14 (67)	27 (26)		
UCLA Loneliness Scale, *M (SD)*	49.5 (11.3)	37.0 (8.8)	5.634	<.001

**Table:** (a) Independent variables included in regression analyses: PTSD (PCL total score)

	Independent variables	Dependent variable PTSD (PCL total score)			
		Estimate	Exponentiated estimate	*t*	*p*
Demographics	Age	−0.012	0.988	−2.43	.017
	Gender	0.062	1.064	0.72	.472
	Race	−0.098	0.907	−1.00	.318
	HIV status	−0.098	1.053	0.72	.475

ACT UP	Years involved in ACT UP	0.004	1.005	0.74	.464
	On a committee	−0.141	0.868	−2.10	.038
	Arrested during demonstration	−0.117	0.889	−1.11	.270

AIDS related	Friends died	−0.027	0.974	−0.21	.834
	Partner died	0.137	1.147	1.63	.107

Past year	Has intrusive thoughts	0.295	1.344	3.71	<.001
	Lacks future orientation	0.171	1.186	2.19	.031
	Feels AIDS is behind them	0.005	1.005	0.06	.952

**Table:** (b) Independent variables included in 2 regression analyses: depression (PHQ total score) and coping (CESD total score)

	Independent variables	Dependent variables						
		Depression (PHQ total score)				Coping (CESD total score)		
		Estimate	Exponentiated estimate^*∗*^	*t*	*p*	Estimate	*t*	*p*
Demographics	Age	−0.013	0.987	−1.09	.278	0.525	1.78	.077
	Gender	0.003	1.003	0.01	.989	2.015	0.39	.694
	Race	−0.278	0.758	−1.27	.205	13.769	2.49	.014
	HIV status	0.122	1.130	0.74	.462	5.725	1.36	.176

ACT UP	Years involved in ACT UP	0.013	1.013	0.95	.343	−0.632	−1.77	.079
	On a committee	−0.288	0.750	−1.83	.069	6.230	1.56	.121

AIDS related	Friends died	−0.133	0.876	−0.44	.658	14.275	1.88	.062
	Partner died	0.334	1.396	1.82	.071	−0.688	−0.15	.882

Past year	Has stable career	0.019	1.019	0.08	.936	1.697	0.28	.780
	Has close friends	−0.716	0.488	−3.92	<.001	12.265	2.64	.009
	In a relationship	−0.283	0.753	−1.37	.174	17.102	3.25	.002
	Part of a community	0.153	1.166	0.72	.474	2.155	0.40	.691

^*∗*^Regression for depression was run with log-normal data; therefore, exponentiated estimates are presented.

**(a):** 

ACT UP experiences	HIV+ men *N* = 41	HIV− men *N* = 61	*t* or *x*^2^	*p*
Age when joined ACT UP, *M (SD)*	29.0 (6.1)	28.7 (5.3)	.292	.771
Number of years in ACT UP, *M (SD)*	6.4 (6.1)	5.1 (4.3)	1.198	.234
Demonstrations, *N (%)*			3.594	.309
None	1 (2)	1 (2)		
A few	0 (0)	1 (2)		
Some	6 (15)	3 (5)		
Many	34 (83)	56 (92)		
Times arrested, *N (%)*			1.418	.492
None	14 (34)	18 (30)		
Once	5 (12)	5 (8)		
More than once	19 (46)	37 (61)		
Attended Monday meetings, *N (%)*			.747	.688
Occasionally	1 (2)	2 (3)		
Often	-	1 (2)		
Regularly	40 (98)	58 (95)		
In a committee, *N (%)*	36 (88)	49 (80)	.987	.320
Lost partner, *N (%)*	16 (39)	11 (18)	5.324	.021
Primary caregiver for sick friend(s), *N (%)*	17 (41)	16 (26)	2.600	.107

**(b):** 

Self-reported measures				
PHQ-9 [depression], *M (SD)*	6.93 (6.93)	4.62 (3.77)	2.205	.03
PHQ-9: DSM IV meets algorithm for major depression, *N (%)*	9 (22)	1 (2)	16.3	<.001
PCL Checklist (PTSD), *M (SD)*	35.3 (15.81)	32.9 (11.4)	1.321	.190
PCL Checklist (PTSD): prorated total minus three depression items, *M (SD)*	34.5 (15.9)	31.3 (11.3)	1.178	.242
AUDIT: problematic alcohol use (scores ≥ 16), *N (%)*	1 (2)	5 (8)	4.13	.127
Coping Self-Efficacy Scale, *M (SD)*	90.5 (26.75)	84.3 (18.7)	1.353	.179
LOT [optimism versus pessimism], *N (%)*			3.911	.141
Optimistic (Scores ≥ 17)	26 (63)	40 (66)		
Pessimistic (Scores < 17)	15 (37)	21 (34)		
UCLA Loneliness Scale, *M (SD)*	19.36 (5.73)	20.5 (4.92)	−.972	.333

**Table 6:** Major categories of response to the question, “Do you think being in ACT UP changed your sense of self, who you are as a person?”

Agency

I found my sense of agency in ACT UP. I found out who I was. I became who I could be because of ACT UP. It transformed me. I developed human skills and brain skills. It made me who I am.
Being in ACT UP changed the epidemic from something that was happening TO me, to something I could do about it. It was a really formative moment in my world framework. People I connected with inspired me to think anything is possible. I have political agency, can fight for beliefs. I was proud of being part of this group.
ACT UP was the most important thing I've ever done in my life. Those years were the least selfish, the most community-motivated. I also learned you never accept authority at the cost of discounting your own experience. It gave me a sense of purpose, of community, of hope - that something could be done.
After ACT UP, I felt able to change things
ACT UP made me step out of myself. I was shy and ACT UP gave me a voice. I felt I could make a change. I spoke from my heart, I did TV. I hadn't known I could organize and lead.

Empowerment

It enhanced my sense of self-worth. I feel compelled to do something when I see something wrong. I feel responsible. I feel I need to improve the lot of others
I found my voice. I learned to speak up for myself and what was right. For the first time I learned to think critically. It was a huge boost to my self-esteem. I felt really proud of what I was doing. Finding my voice, finding I had something to give and effect change and I had a part in doing something that changed the world.
It changed my feelings about myself. It made me more confident, more outgoing. I was really fearless back then in my 20's. Even if I never find it, I keep searching for the sense of purpose I had in ACT UP.
The experience changed my outlook, my sense of what I could accomplish, my view of the world, view of myself. It taught me I could do things I had no idea I could do. It taught me there were other people like me: smart, spiky, angry, fiercely committed. We had an effect.
It gave me confidence both socially and in my ability to motivate people. It felt empowering, being involved in something meaningful. I felt empowered we were going to save our friends' lives, inspired by the combination of street smarts and intelligence.

Personal growth

Being a member of a group working at a high level, knowing I could operate at that level. Discovering in myself powers I didn't know I had.
It made me much more confident and capable. I ended up more willing to try new things and try different directions. The skills I learned at ACT UP stayed with me. I believe in gay community and the importance of gay community, a bedrock belief that being gay matters, and I learned that at ACT UP.
It brought meaning to my life. It helped define, was part of my identity. I was proud to be part of ACT UP. It gave me a sense of purposefulness, feeling of use, self-worth. Now I can speak in front of a group of people and have the courage to say what I think.
It made me feel a better person, more serious, smarter, stronger.
Helped me define myself. I learned to respect that I have a voice, have my opinions, can work with others. Before, I didn't think people listened to me. I no longer felt like a contagious person. It changed my thinking about myself.

## Appendix

Response options to the question, “In what ways was ACT UP a positive experience?”

1. Sense of empowerment vis-à-vis AIDS
2. New or stronger social ties (with others in ACT UP)
3. Personal recognition, leadership role (limelight)
4. Sense of collective identity, morale
5. Fighting for one's life (or one's community's survival)
6. Connecting with the gay community/confirming identity
7. Sexual networking
8. Emotional/psychological support
9. Success in developing trials and treatments
10. Stimulating/educational about HIV treatments

Response options to the question, “Since leaving ACT UP, do you miss anything?”

1. Feelings of irrelevance
2. Loss of direction
3. Loss of a cause, or doing something that matters
4. Loss of ACT UP limelight, early fame
5. Loss of network
6. Loneliness
